# Immediate Effect of Simulated High Heels on Pelvic and Spinal Posture in Healthy Young Subjects: A Cross-Sectional Study

**DOI:** 10.7759/cureus.55586

**Published:** 2024-03-05

**Authors:** Saverio Colonna, Corrado Borghi, Matteo Galvani, Antonio D'Alessandro

**Affiliations:** 1 Osteopathic Spine Center Education (OSCE), Spine Center, Bologna, ITA

**Keywords:** kiphotic angle, lordotic angle, pelvic inclination, trunk inclination, raster-stereography, spine posture, high-heels

## Abstract

Background

Investigations regarding the role of high-heeled shoes in the alteration of the spinopelvic profile attempted to identify a correlation with pain in the lower back. Conclusions from these studies, however, are controversial. In authors knowledge no studies were carried out to investigate the effect of heels on male population, which has been overlooked due to gender-related customs.

Research question

What is the immediate effect of the height of heels on the sagittal back profile (trunk inclination (TI), pelvic inclination, lordotic lumbar angle (ITL-ILS), kyphotic dorsal angle, lumbar arrow, and cervical arrow) in females and males, not used to wearing high-heeled shoes?

Methods

One hundred healthy young adult subjects were enrolled. Three were excluded. The remaining 97 subjects (48 female and 49 male) underwent a three-dimensional analysis of the posterior surface of the trunk, using rasterstereography. The spinopelvic profile in the barefoot condition, and with the heel raised by 3 and 7 cm, was recorded. To evaluate the reproducibility of the measure, the neutral evaluation was repeated twice in 23 subjects (13 males, 10 females).

Results

The change of heel height did not show statistically significant differences for any of the variables used; instead, significant differences were found stratifying the results according to the sex of the subjects tested. Test-retest evaluation in the neutral condition showed no significant differences using the Student's t-test (p > 0.05). Repeatability was excellent and significant for all data used (minimum TI r = 0.85, maximum ITL-ILS r = 0.97).

Significance

Studying the effect of heels on the spino-pelvic profile also in the male population is crucial for promoting gender-inclusive healthcare, enhancing occupational health practices and developing possible preventive measures. Nevertheless, in the sample of females and males evaluated in this study, the different heights of heel lift did not immediately induce significant changes in pelvis and spine posture. If there is therefore a correlation between low-back pain and the use of heels, it should not reasonably be sought in the immediate change of the spino-pelvic profile caused by raising the heels. However, the variables analyzed differed according to sex.

## Introduction

High heeled shoes are frequently used to look taller, more elegant, and attractive. However, some authors claim that women who often use higher heels run a greater risk of back pain [1-3]. This reported presence of pain has led to the study of alterations in the spino-pelvic profile to search for possible causes. Investigations regarding the role of high-heeled shoes in the alteration of the spino-pelvic profile have attempted to explore compensatory and adaptive structural changes as the reason for pain in the lower back. Conclusions from these studies, however, are controversial. Some studies [4-6] have shown that high heeled shoes greatly increase lumbar lordosis (LL), and consequently mechanical load and degenerative changes in the soft tissues [4]. Other studies [7,8] found a reduction in LL and a compensatory increased activity of the erector spinal muscle [9]. The results of other works indicated that the heel did not significantly modify the LL in the subjects evaluated [1,10-13].

Oliveira Pezzan et al. [9] found different behaviors in frequent and non-frequent high-heel users. They discovered an increase in pelvic anteversion and LL only for frequent users, whereas the effect in the other group was a decrease in pelvic tilt and lordosis.

In authors knowledge no studies were carried out to investigate the effect of heels on male population. There are numerous publications about the human lumbar lordosis: about half of the studies found no statistical difference between males and females and the other half found that females have significantly greater lordosis angles (2-5°) difference [14]. For Hay et al. [15] males and females manifest different lumbar curve shape, yet similar amount of inward curving (lordosis). Stagnara et al. [16] suggested that females apparently had greater lumbar lordosis owing to their greater buttock size.

The purpose of this study, given the considerable heterogeneity of data in the literature, is to investigate whether artificial heel lift affects immediate spinal posture and pelvic position in the sagittal plane in a group of healthy men and women. Studying the effect of heels on the spino-pelvic profile in the male population is important for several reasons. Ensuring that both genders receive adequate attention in research and healthcare is essential to promote gender equality. Investigating the impact of heels in certain professions, such as theater, fashion, or entertainment, where men may wear heels as part of their costumes or roles, is relevant for occupational health issues. For both genders, a better understanding of the spine's immediate response during heel lift could increase knowledge in understanding the hypothetical association of low-back pain with heels and help preventing and/or treating it. This could include footwear design modifications, ergonomic practices, or specific exercises to alleviate strain on the back muscles.

## Materials and methods

Study design

The present investigation is a monocentric cross-sectional study (ClinicalTrials registration number NCT05593991). Participants went through four measures in different conditions: neutral (baseline), performed twice to determine the repeatability of the evaluation system; heel raised by inserting a 3 cm and then 7 cm plastic support symmetrically under both heels.

Subjects

One hundred healthy adults, unaccustomed to wearing high-heeled shoes, were enrolled. All of them were recruited in the OSCE (Osteopathic Spine Center Education) school of osteopathy based in Bologna, Italy, through announcements during lessons and on the bulletin board.

Subjects with the following characteristics were excluded: structural or neurological pathologies that would prevent the maintenance of an upright posture for 5 seconds on a 7 cm heel support; back pain in the last 30 days. The number of female subjects to be evaluated was decided based on the most recent works, which analyzed respectively 36 [12] and 56 [17] women. A similar number between males and females was designed to improve the statistical comparison. Sex was considered as the one assigned at birth.

Instruments

The gold standard for assessing the sagittal and frontal balance of the spine, used for example in scoliosis, is X-rays [18]. However, the radiation produced by this method is a major drawback, especially in young patients [18]. An alternative but precise, radiation-free, and inexpensive method of detecting spinal posture, especially for follow-up, is rasterstereography [19-21]. Surface analysis is performed by projecting a light grid modeled onto the patient's back. Anatomical landmarks can be detected automatically by assigning concave and convex areas. By detecting predetermined anatomical points, the system is able to calculate a three-dimensional model of the human spine. From this model relevant clinical parameters can be determined, such as: pelvic obliquity, trunk inclination, kyphosis and lordosis angles [22-24 ]. In this study the rasterstereographic device used was the Formetric 4D® (Diers International GmbH, Schlangenbad, Germany).

**Figure 1:**
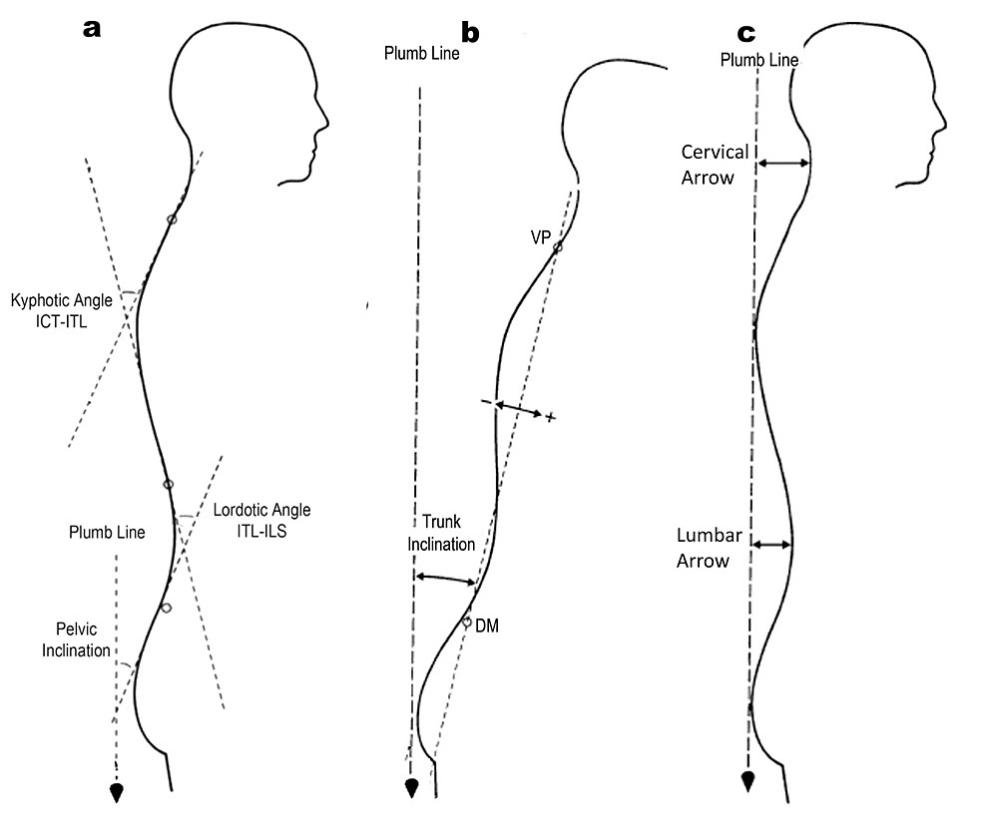
Data collected from the rasterstereographic device a) Pelvic Inclination angle (PI), Lordotic angle (ITL-ILS), Kyphotic angle (ICT-ITL); b) Trunk Inclination (TI); c) Lumbar arrow (LA); Cervical arrow (CA). ITL: Thoracolumbar junction; ILS: Lumbosacral junction; ICT: Cervicothoracic junction. Image credits: Colonna S.

**Figure 2:**
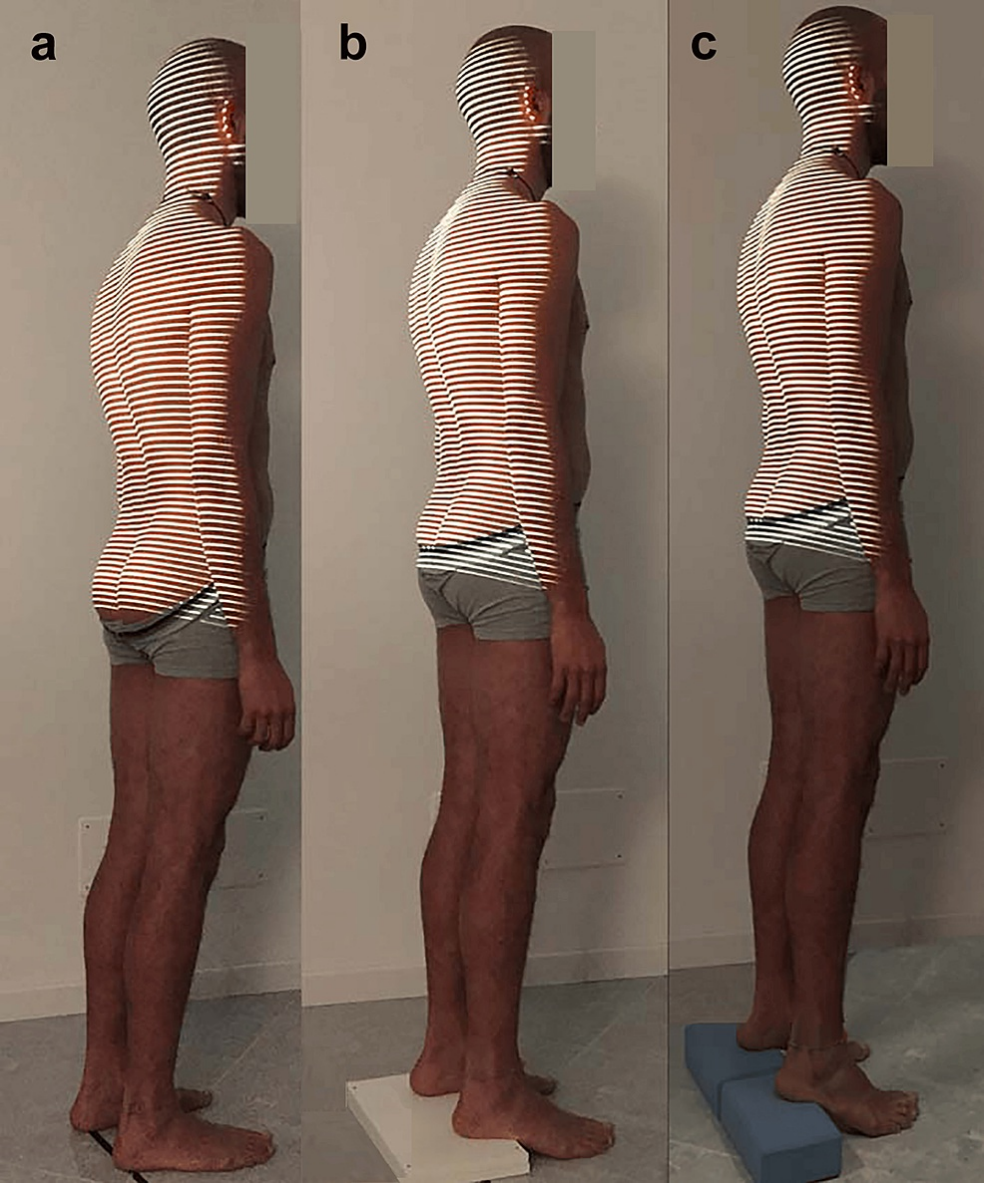
Assessment conditions a) Barefoot; b) Heels raised 3 cm; c) Heels raised 7 cm. Image credits: Colonna S.

Data collected were: 1) antero-posterior trunk flexion (Trunk Inclination (TI)) measured as the angle between the plumb line and the line passing through the prominent cervical vertebra (VP) to the line that connects the two dimples (DM), Figure 1b; 2) pelvic inclination angle (PI), the angle spanned by the vertical and the tangent lumbosacral junction (ILS); 3) lordotic angle ITL-ILS, measured between the tangents of the thoracolumbar junction (ITL) and the lumbosacral junction (ILS); 4) kyphotic angle ICT-ITL, measured between the tangents of the cervicothoracic junction (ICT) and the ITL (Figure 1a); 5) lumbar arrow (LA; horizontal distance in millimeters from the virtual vertical plumb line passing through the kyphotic apex in the lumbar spine); 6) cervical arrow (CA; horizontal distance in millimeters from the virtual vertical plumb line passing through the kyphotic apex in the cervical spine; Figure 1c).

Participants provided information about age, height, and weight. A questionnaire was given to standardize the types of high-heeled shoes used and to verify their frequency of use. Further information was collected regarding the frequency of weekly use and the number of hours spent wearing the high-heel shoes.

Evaluation protocol

Subjects were prepared so that their back was clearly visible from the hairline to the sacrum (Figure 2), without rings, watches, and necklaces that could interfere with the measure. The data were collected while subjects were standing in a relaxed posture, with fully extended knees and arms naturally dangling over the hips. To standardize the position of the subjects, adhesive tape was positioned on the floor to provide a reference for the subjects’ feet. Positioning with respect to the measurement system was carried out according to the recommendations of the supplier. Measures were performed in the following order: (1) first evaluation in a neutral position barefoot; (2) second evaluation in a neutral position barefoot; (3) 3-cm support below both heels; (4) 7-cm support below both heels.

The first two evaluations were conducted to assess the reproducibility of the data. The time between the two evaluations was less than one minute and the subject did not change position. After two minutes the heel was raised by inserting a plastic support, as proposed in previous studies where plastic or wood was used [8,17,25]. Raster-stereographic analysis was always performed by the same two skilled operators, a third operator then transcribed the results on an Excel spreadsheet and a fourth operator performed the statistical analysis.

Statistics

Descriptive analysis was used to determine the mean values and standard deviations of the tested population. Shapiro-Wilk test was applied to verify the normal distribution. Intra-class correlation coefficient (ICC) test and paired t-test was utilized to assess repeatability of data between first and second observation in neutral condition. The result was interpreted according to the guidelines for ICC inter-rater agreement measures: less than 0.40-poor; between 0.40 and 0.59-fair; between 0.60 and 0.74-good; between 0.75 and 1.00-excellent [26]. One-way ANOVA was used for comparison of neutral position, heels lifted 3 cm and 7 cm in the full group (females plus males) and stratified by sex (only females and only males). In case of significant ANOVA differences between the different positions (0 cm - 3 cm - 7 cm), the Bonferroni post hoc for dependent data was applied. Two-way ANOVA was performed considering heel and sex simultaneously. Statistical significance was set with a p-value ≤ 0.05. Data analysis was performed using R version 3.5.1 (The R Foundation for Statistical Computing, Indianapolis, IN, USA) software.

## Results

Three of the 100 subjects enrolled were excluded due to pain/discomfort in the sole of the foot (two female subjects) and lumbar pain/discomfort (one male subject) which did not allow for completing the 7 cm elevation evaluation; hence, 48 females (mean age 28.8 ± 8.0, weight 57.3 ± 7.38 kg, height 166 ± 6.6 cm) and 49 males (mean age 29.1 ± 8.1, weight 74.3 ± 8.5 kg, height 178.8 ± 5.5 cm) were analyzed.

The evaluation of the data obtained using the Shapiro-Wilk test highlighted that in only two of the parameters detected (ITL-ILS, LA) there was acceptance of normal distribution (p > 0.05) (Table 1); anyway, by comparing graphically the parameters investigated with the normal, it is possible to note, for most of the variables, a distribution with a slightly asymmetric unimodal trend. For this reason and aware that the underlying error we would encounter using all the data obtained as non-parametric would be greater, it was decided to continue to treat the data as if they were all normally distributed, accepting the error to be encountered.

**Table 1:** Normality distribution of variables and results of the two-way ANOVA considering the height of the heel lift and the sex. TI: Trunk Inclination; PI: Pelvic Inclination angle, ICT-ITL: Kyphotic angle; ITL-ILS: Lordotic angle; CA: Cervical arrow; LA: Lumbar arrow; ITL: Thoracolumbar junction; ILS: Lumbosacral junction; ICT: Cervicothoracic junction. According to the Shapiro-Wilk test, the variables have a normal distribution (acceptance) or don't have a normal distribution (rejection).

	NORMALITY DISTRIBUTION		TWO-WAY ANOVA	
VARIABLE	P-VALUE	RESULT	P-VALUE HEEL HEIGHT	P-VALUE SEX
TI	< 0.001	rejection	0.298	0.002
PI	0.036	rejection	0.109	< 0.001
ICT-ITL	< 0.001	rejection	0.409	0.584
ITL-ILS	0.065	acceptance	0.678	< 0.001
CA	< 0.001	rejection	0.815	< 0.001
LA	0.055	acceptance	0.129	0.839

Reliability of the data expressed in the test-retest in neutral position, using the Student's t-test (p <0.05), did not show significant differences, instead it demonstrated an excellent repeatability (ICC) significant for all data used (minimum TI ICC = 0.85, maximum ITL-IL ICC = 0.97).

Table 2 shows the means and standard deviations of the results. The analysis of the differences among the evaluations at 0 cm (neutral), 3 cm and 7 cm, by ANOVA, did not show any significant differences for the parameters analyzed, both considering all subjects (males + females) and stratifying the group by the sex. Since no significant differences were found, the use of Bonferroni's post hoc analysis for dependent data was omitted.

**Table 2:** Mean and standard deviation of the analyzed variables TI: Trunk Inclination; PI: Pelvic Inclination angle; ICT-ITL: Kyphotic angle; ITL-ILS: Lordotic angle; CA: Cervical arrow; LA: Lumbar arrow; ITL: Thoracolumbar junction; ILS: Lumbosacral junction; ICT: Cervicothoracic junction. Data are reported in degrees for TI, PI, ICT-ITL and ITL-ILS, in mm for CA and LA.

VARIABLE	HEEL HEIGHT [mm]	ALL SUBJECTS	FEMALE	MALE
TI	0	2.3 ± 2.0	1.8 ± 1.7	2.8 ± 2.2
	3	2.1 ± 2.2	1.7 ± 1.8	2.6 ± 2.6
	7	2.0 ± 2.1	1.8 ± 1.6	2.2 ± 2.5
PI	0	20.6 ± 6.8	25.3 ± 5.2	16.1 ± 5.1
	3	20.1 ± 6.9	24.5 ± 4.8	15.9 ± 6.0
	7	19.4 ± 6.9	24.4 ± 4.7	14.6 ± 5.1
ICT-ITL	0	46.9 ± 9.3	47.4 ± 9.3	46.6 ± 9.4
	3	47.9 ± 9.1	48.4 ± 8.9	47.7 ± 9.6
	7	48.1 ± 9.1	48.3 ± 8.5	48 ± 9.8
ITL-ILS	0	40.9 ± 10.4	47.1 ± 9.4	35 ± 7.7
	3	40.9 ± 10.3	47.2 ± 8.8	34.8 ± 9.6
	7	40.4 ± 10.4	46.7 ± 9.2	34.5 ± 7.7
CA	0	59.7 ± 18.0	49.7 ± 12.3	69.7 ± 17.4
	3	60.7 ± 17.8	50.9.4 ± 12.0	70.5 ± 17.3
	7	60.4 ± 18.1	51.0 ± 12.5	69.6 ± 18.2
LA	0	39.6 ± 12.1	40.3 ± 11.8	39.3 ± 12.7
	3	41.6 ± 12.6	42.2 ± 12.5	41.4 ± 12.7
	7	42.5 ± 12.5	42.2 ± 11.7	43 ± 13.6

**Figure 3:**
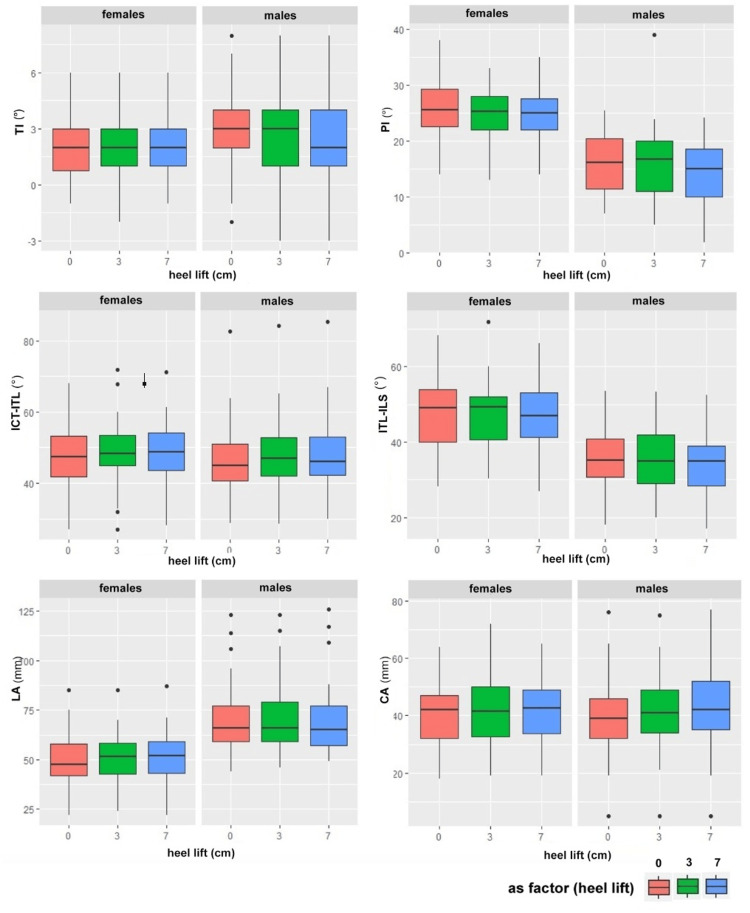
Results stratified by heel and sex Graphical box-plot presentation of the results where the stratification by heel and sex of the analyzed variables is shown. TI: Trunk Inclination (top left); PI: Pelvic Inclination angle (top right); ICT-ITL: Kyphotic angle (middle left); ITL-ILS: Lordotic angle (middle right);  LA: Lumbar arrow (bottom left); CA: Cervical arrow (bottom right); ITL: Thoracolumbar junction; ILS: Lumbosacral junction; ICT: Cervicothoracic junction.

The interpretation of the two-way ANOVA, which jointly considers the presence of heel and sex, shows that heel length is not significant, while sex is significant (p < 0.05) for the variables TI, PI, ITL-ILS, and CA (Table 1). In Figure 3, the boxplots highlight the relationship between heel lift, height, and sex.

**Figure 4:**
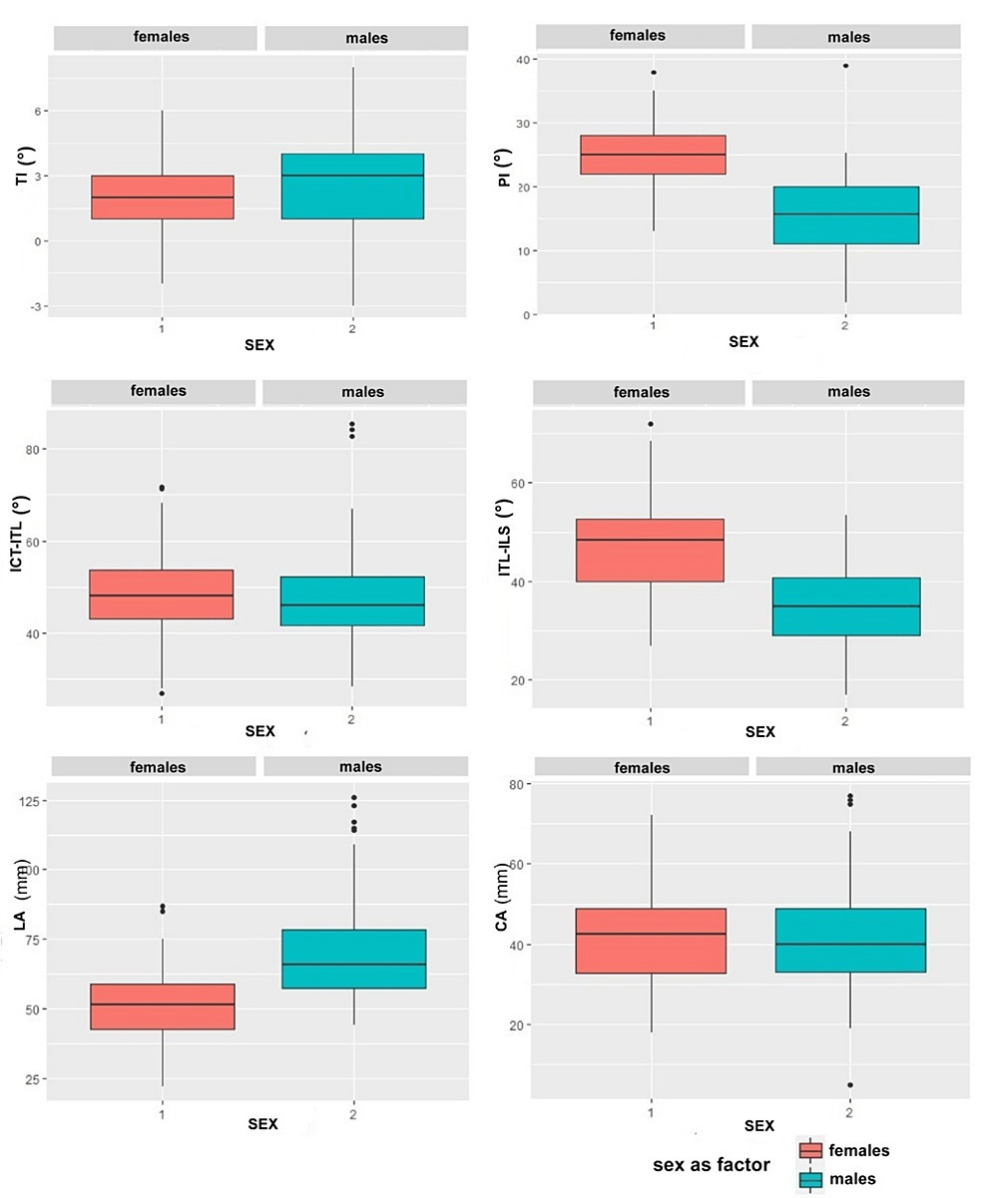
Results stratified by sex Graphical box-plot presentation of the results where the stratification by sex of the analyzed variables is shown. TI: Trunk Inclination (top left); PI: Pelvic Inclination angle (top right); ICT-ITL: Kyphotic angle (middle left); ITL-ILS: Lordotic angle (middle right);  LA: Lumbar arrow (bottom left); CA: Cervical arrow (bottom right); ITL: Thoracolumbar junction; ILS: Lumbosacral junction; ICT: Cervicothoracic junction.

The two-way ANOVA indicates a significant difference only with regard to sex. TI shows a wider variability in males, with a higher median value than in females; males show a lower median of PI; ICT-ITL has a minimal difference between males and females, not significant, with males having a slightly lower median than females; ITL-ILS has a lower median in males than the females; the median value of CA observed in males is greater than in females; LA does not show significant differences, suggesting that both heel and sex are not significant variables in explaining the response of this parameter. Figure 4 shows the graphic representation of the result according to the sex.

## Discussion

The purpose of this study was to investigate whether artificial heel lift immediately affects the spino-pelvic profile in the sagittal plane in a group of healthy men and women. The evaluation of the distribution of our data using the Shapiro-Wilk test appears to be normal in only two of the parameters detected (ITL-ILS, LA); this is in contradiction with the data reported in the literature by another study [12] which used the same evaluation tool (Formetric®-System) but with Kolmogorov-Smirnov with a significance level of p > 0.10. The authors' choice to use a significance level of p > 0.10 (90%) for analyzing the normality of the distribution, as opposed to other methods like the t-test which typically use a level of p < 0.05 (95%), remains unclear. We can only speculate that facing similar circumstances, lowering the significance level allowed them to achieve normal distribution for all variables, enabling them to proceed with parametric tests. However, using non-parametric tests in such cases may reduce the precision of the evaluation.

The test-retest in neutral position showed excellent repeatability of the rasterstereography in the evaluation of the sagittal profile, thus confirming the data presented in the literature [27-29]. The data obtained from our study regarding TI suggested a general extension of the trunk in line with the work of [17], but our results did not reach statistical significance. This disagrees with the findings of Dai et al. [6] and Drzał-Grabiec and Snela [13] which showed an increase in SVA (Sagittal Vertical Axis), with a C7 anterior translation with respect to the sacrum.

The change in pelvic tilt doesn't achieve statistical significance, consistent with findings from certain authors [4,6,7,13]; however, other studies report retroversion [8,12,17,30]. Conversely, De Oliveira Pezzan et al. discovered retroversion solely among individuals unaccustomed to wearing high heels, while those accustomed to them exhibited anteversion [9].

According to Bendix et al. [25], these changes occurred because of a trunk displacement adopted to reduce the anterior body tilt perceptions and the tendency to hyperextend the hip due to the posterior pelvic tilt. The pelvis is considered a key structure of body alignment, and any changes in its neutral position will cause compensatory movements in various regions, with the lumbar spine being one of the most affected segments.

Concerning the dorsal kyphosis evaluation, our data are indicative of an increase in the representative angle of this spinal curve, but without reaching statistical significance, both considering the single group, only females or only males. In the literature there are few papers that have evaluated the relationship between the elevation of the heel and kyphosis. Our data are in line with the results of Michoński et al. [17] and Drzał-Grabiec and Snela [13] and they are different from those of Dai et al. [6] in which 15 (71.43 %) of the 21 participants had an increase of 3.4° in the thoracic kyphosis value. In our analysis, the average increased angle, in addition to not reaching the significant level, was also only 1°, so its clinical relevance is questionable. The lack of significant difference in dorsal kyphosis in our study should not come as a surprise since it closely correlates with lordosis and follows its pattern [6,17].

The ICT-ITL outcomes are indicative of a trend towards a reduction in the lordotic curve, despite not reaching statistical significance. This trend confirms the findings of other study who used different lordosis detection methods [1,7,10,11,13,17]. For the evaluation of the arrows variable, only the result of the lumbar arrow tends to increase together with the increase in heel height both for the groups divided by sex and globally together, without reaching statistical significance. To our knowledge, there are no studies in the literature that have used these variables to evaluate the reaction of the spine profile to changes in heel height.

Understanding the discrepancies in the literature regarding the effects of heel elevation on the spino-pelvic profile, ranging from increased lordosis in some studies to no change or even decreased lordosis in others, poses a challenge. Possible explanations may include factors such as age, the frequency of wearing high-heeled shoes, variations in methodologies for evaluating spinopelvic parameters, and differences in heel elevation techniques. For example, conflicting findings exist regarding lumbar lordosis, with studies showing that the impact of high-heeled footwear depends on participants' familiarity with wearing heels and their age [5,9,25,30]. Some researchers concluded that increased lumbar lordosis angles were more prevalent among inexperienced users, while others found increased lordosis in women accustomed to wearing high heels [5]. Furthermore, methodological differences across studies further complicate comparisons. While some researchers focus on static outcome parameters [4,6,7,9,11,12,17,25,30], others examine dynamic outcomes [4,6,12]. Additionally, assessment tools vary, including low-dose whole-body X-ray [6], indirect back surface reconstruction [11], lateral photogrammetry [7], spinal mouse [11], and rasterstereography [12].

To our knowledge, this is also the first study that compares the immediate spine reaction to heels in males and females. It is possible that the differences in the conformation of the spino-pelvic structures of the two sexes lead to a different response, in terms of lower back pain, from the raising of the heel. Many studies have debated whether differences exist between male and female spinal architecture in neutral conditions, and several sagittal X-ray studies measuring lordosis differences have been conducted. Vialle et al. [31] observed a difference in lumbar lordosis in males and females (larger lordosis in females as measured by the Cobb angle). Similarly, based on a 3-D electromechanical digitizer to derive curvature angles for the region of the spine between T12-L1 and S2, Norton et al. [32] found that the lordosis angle (calculated using the ratio between the lordosis depth and length) was 13.2° larger for women than for men. On the other hand, a study conducted using the Cobb angle measurement on plain radiographs (X-rays taken of individuals in a standing position) of an asymptomatic Greek population, demonstrated that thoracic kyphosis and lumbar lordosis (T12-S1, L1-L5) were not sex-related [33]. A study conducted on 60 healthy males and females using innovative upright low-dose digital biplanar X-rays and three-dimensional analysis [32] also failed to reveal a significant difference in lordosis between males and females. The study also examined the sagittal spinal inclination defined as the angle between a vertical and best-fit straight line passing through the centroids of the vertebrae and found that the thoracic and thoracolumbar vertebrae were more dorsally (backwardly) inclined in women than in men. Bailey et al. [34] found a lordotic curve in females significantly greater (7.3°) compared to males, but only in the orthostatic position. However, in the supine condition, there was no significant difference.

In this study, the results confirm a sex-related difference for the three conditions, which reach the required level of significance (p < 0.05): greater trunk inclination (TI) in males; less pelvic tilt (PI) and lumbar lordosis (ITL-ILS) in males; greater cervical arrow (CA) in males. The data about the neutral condition agree with the findings of Vialle et al. [31], Norton et al. [32], and Bailey et al. [34].

Limitations

The first limitation of this study is the lack of pain assessment. As with other studies in the literature [1,6,9,12,25], the aim was to conduct an initial phase of investigation of the pelvis and spine's behavior when the foot support is altered. This serves as the foundation for establishing a rationale linking that behavior to pain. Another critical issue is how different heel heights were achieved. Of course, the support used was different from real heels; therefore, further research needs to be performed to highlight spine and pelvis changes due to real high-heeled shoes.

The third sticking point is the time duration the heel was lifted. Usually, complaint of back pain occurs after wearing high heels for a long time and not immediately after wearing them. It is possible that the muscles, which initially manage to compensate without changing posture as the heel rises, suffer from fatigue, and the curves analyzed can become dysfunctional after a period of time, leading to overloads that are a source of inflammation and pain. In future studies it would be recommended to evaluate the heels effect both immediately and after an adjustment time, to control the relevance of vertebral muscle fatigue.

The assessment using rasterstereography demonstrates excellent reliability in evaluating spino-pelvic parameters on the sagittal plane [29], as confirmed by both our test and retest. However, its reliability significantly diminishes in the axial and coronal planes [35]. Since our study solely focused on sagittal parameters, the limitations of the equipment should not have substantially influenced the results.

## Conclusions

In the group of females evaluated in this study, different heel heights did not appear to immediately induce a significant change in pelvic and spinal posture. An identical trend is present in the male group. A significant difference between males and females is confirmed in most of the examined parameters (TI, IP, ITL-ILS, CA) regardless of heel height. There was no significant effect on immediate change on the spino-pelvic profile that could justify repercussions on lumbar disorders. These findings are valid both for males and females. Hypothetical correlations between heels, spino-pelvic profile modifications, and back pain should be investigated by future studies considering the muscle fatigue produced by prolonged high-heel use. Rasterstereography, a radiation-free method used to determine body posture in the sagittal plane at different levels, was confirmed to be a reliable method of examination of the sagittal back profile.
